# An Integrated SNP Mining and Utilization (ISMU) Pipeline for Next Generation Sequencing Data

**DOI:** 10.1371/journal.pone.0101754

**Published:** 2014-07-08

**Authors:** Sarwar Azam, Abhishek Rathore, Trushar M. Shah, Mohan Telluri, BhanuPrakash Amindala, Pradeep Ruperao, Mohan A. V. S. K. Katta, Rajeev K. Varshney

**Affiliations:** 1 Centre of Excellence in Genomics, International Crops Research Institute for the Semi-Arid Tropics (ICRISAT), Patancheru, India; 2 School of Agriculture and Food Sciences, University of Queensland, Brisbane, Australia; National Institute of Plant Genome Research, India

## Abstract

Open source single nucleotide polymorphism (SNP) discovery pipelines for next generation sequencing data commonly requires working knowledge of command line interface, massive computational resources and expertise which is a daunting task for biologists. Further, the SNP information generated may not be readily used for downstream processes such as genotyping. Hence, a comprehensive pipeline has been developed by integrating several open source next generation sequencing (NGS) tools along with a graphical user interface called Integrated SNP Mining and Utilization (ISMU) for SNP discovery and their utilization by developing genotyping assays. The pipeline features functionalities such as pre-processing of raw data, integration of open source alignment tools (Bowtie2, BWA, Maq, NovoAlign and SOAP2), SNP prediction (SAMtools/SOAPsnp/CNS2snp and CbCC) methods and interfaces for developing genotyping assays. The pipeline outputs a list of high quality SNPs between all pairwise combinations of genotypes analyzed, in addition to the reference genome/sequence. Visualization tools (Tablet and Flapjack) integrated into the pipeline enable inspection of the alignment and errors, if any. The pipeline also provides a confidence score or polymorphism information content value with flanking sequences for identified SNPs in standard format required for developing marker genotyping (KASP and Golden Gate) assays. The pipeline enables users to process a range of NGS datasets such as whole genome re-sequencing, restriction site associated DNA sequencing and transcriptome sequencing data at a fast speed. The pipeline is very useful for plant genetics and breeding community with no computational expertise in order to discover SNPs and utilize in genomics, genetics and breeding studies. The pipeline has been parallelized to process huge datasets of next generation sequencing. It has been developed in Java language and is available at http://hpc.icrisat.cgiar.org/ISMU as a standalone free software.

## Introduction

Next Generation Sequencing (NGS) technology has changed the research landscape of genomics especially crop genomics since the last few years [1]–[4]. Illumina, Life Technologies' SOLiD and Ion Torrent sequencing platforms have been used for rapidly identifying SNPs and other marker studies in crops. These platforms usually produce short reads of 50–150 bp which have been preferred for SNP identification. Rapid technological advances in recent times enabled NGS technologies to provide significantly higher throughput at lower cost. However, the cost involved in sequencing experiments is very high, especially, for species with large genomes or species for which the reference genome is not available (orphan crops). In such cases, transcriptome sequencing from more than one genotype is a first choice for plant genetics and molecular breeding community to decipher SNPs. The advantage of this approach is that the identified SNPs are mostly located in single copy genes, which are a pre-requisite for SNP marker analysis. In various model or major crop species, transcriptome sequencing has been used for allele discovery and gene expression analysis [5]–[9]. Here, the limited number of SNPs detected in the genes was due to selection constraints in coding regions which result in finding only few thousand useful markers. To overcome this limitation, alternative approaches have been developed. These approaches use NGS technologies in combination with complexity reduction technologies [10]. These complexity reduction technologies cover the entire genome not limited to protein coding regions. Hence, other single-copy sequences can be surveyed for SNPs as well. Complexity reduction technologies are based, for example, on the selective sequencing of a DNA fraction derived from the digestion with methylation sensitive restriction enzymes [11]–[14], the pre-amplification with specific AFLP (amplified fragment length polymorphisms) primer combinations [15] or the use of the RAD (restriction-site associated DNA) sequencing technology [16]–[18]. In comparison to the transcriptome-based approach, the complexity reduction technologies have an advantage that they find use more or less independent of the genome size. However, NGS technology has been used recently for sequencing and whole genome re-sequencing projects with an objective to mine for a large number of SNPs towards constructing haplotype maps, exploring diversity within-species and performing genome-wide association studies (GWAS) for trait mapping [19]–[21].

Identified SNPs are highly effective only when they are distributed throughout the genome and genotyped for the population of interest or for a set of germplasm. In general, SNP discovery using NGS is limited to the number of lines assayed or sequenced. Genotyping large number of SNPs facilitates various genetic analyses (e.g. phylogenetic analysis, ultra-dense genetic mapping and genotype/phenotype association studies) and important applications (e.g. cultivar identification, marker-assisted selection). The best approach towards the SNP marker discovery for genotyping is to compare fully sequenced genomes from individuals of a given species. However, in case full reference sequence is not available, reads could be assembled to derive a mimic reference sequence and genome-wide SNPs can be identified [22]. In recent years, several such orphan crops have been enriched with full or partial reference genome sequence information. Comparing the re-sequencing data with the reference genome sequence provides a large number of SNPs throughout the genome and has been described in various crop species such as chickpea [23], maize [24], rice [25], and soybean [26]. Identification of SNPs allows their application as markers in various genetic analyses. These genetic analyses further require genotyping of SNP markers on a full set of the material/population. Even though the genotyping methods are limited by the cost and time for scoring SNPs, there has been a steady development of various high-throughput and low cost automated genotyping platforms. These platforms were primarily developed for human genomics, nevertheless, they have been adopted and used for plant species such as chickpea [23], [27], maize [28], pigeonpea [29], rice [30] and wheat [31]. They are very efficient in handling large number of samples and can genotype one to one million SNPs [32], [33] in parallel. Some popular technologies like TaqMan and SNPlex™ from Applied Biosystem/Life technologies (Carlsbad, CA, USA), array based technologies like GoldenGate and Infinium technology from Illumina (San Diego, CA, USA) and PCR based technology like KASP from LGC limited (Teddington, Middlesex, UK) are the most sought-after options for molecular breeders towards genotyping studies owing to low cost, high-throughput and automated workflow.

In general, SNPs could be utilized to enrich the genomic resources for a species in mainly two steps, first, is to identify DNA sequence polymorphisms (marker discovery) and then, second is to assay the identified SNP markers (genotyping) across the segregating population(s) or germplasm set. In this context, several data analysis pipelines such as ngs-backbone [34], SeqGene [35], Games [36], inGap [37] and GATK [38] have become available that process data stepwise manner to identify SNPs from NGS data. These pipelines vary in the type of input data for SNP analysis. For example, some pipelines start with raw data, some with processed data and some from alignment data. These pipelines in general provide a list of SNPs between the reference genotype and sample. In fact, low concordance is observed amongst the SNP of multiple SNP calling pipelines. These are mainly due to diverse integrated alignment tools and SNP prediction methods. Furthermore, almost all pipelines available as open-source are command line based and work only on Linux platform. Moreover, the identified SNPs need to be processed and filtered further to design assays for using them as marker-genotyping assays. In fact, to the best of our knowledge, the step of producing input file for the filtered and high-quality SNPs for assay designing is not available in any pipeline. Therefore, the present study was undertaken with an overall objective to develop a graphical user interface (GUI) based, automated SNP discovery pipeline from NGS data. We have called it as Integrated SNPs Mining and Utilization (ISMU) pipeline. The pipeline identifies SNPs from the NGS data of samples and extracts informative SNPs for genotyping platforms such as Illumina (BeadXpress, GoldenGate, Infinium) and KASP. ISMU supports single end (SE) as well as paired end (PE) data and lists the informative SNPs with polymorphism information content (PIC) values. To facilitate the analysis in small laboratories with limited computation facilities, the pipeline is distributed on request as a virtual machine image as well as on Compact Disk (CD).

## Results

ISMU is an easy to use GUI based pipeline for SNP discovery useful for experimental biologists, geneticists and plant molecular breeding community. It allows researchers to analyse NGS data generated by Illumina, Life Technologies (SOLiD) or reanalyze already deposited data from SRA (Sequence Read Archive) of NCBI. It is highly customizable and integrates popular command line based tools for alignment and SNP prediction with support for both SE and PE data in a standard FASTQ format [39]. It is designed to run on 64 bit desktop machines. The support for multi-threading in ISMU enables the user to analyze high volume data. The pipeline provides high quality SNPs that could be used for genotyping assay development for KASP and Illumina platform. All the result files are flat text files except a spreadsheet namely “called_allele_data.xls”. The pipeline additionally generates a list of INDELs if BWA/Bowie2 was used for alignment.

### Features of the pipeline

**Figure 1:**
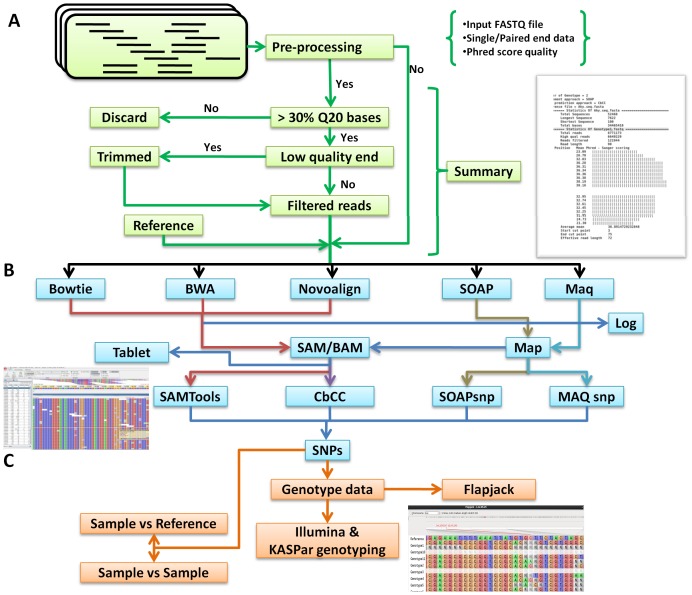
The work-flow of the ISMU pipeline. The work-flow of the ISMU pipeline is mainly divided into three steps: (A) Data import, quality pre-processing, (B) Sequence alignment and SNP discovery, and (C) Visualization and generation of input files for genotyping assay.

Identification of SNPs is a multi-step process involving various tools for pre-processing the data, alignment against the reference and identifies variations subject to filters. The work-flow (Figure 1) could be broadly categorized into the six steps as follows: (i) data import, (ii) data pre-processing, (iii) sequence alignment or mapping short reads onto the reference genome, (iv) SNP discovery, (v) visualization, and (vi) generation of genotyping assay input files. All these steps have been built the automated ISMU pipeline for ease of use. ISMU offers a wide choice of tools at every step with default parameters. Multiple options enable comparison across different (alignment and SNP calling) methodologies and thereby arrive at a concordant set of SNPs for targeted genotyping.

#### Importing the data

The pipeline requires input files in the FASTQ format [40]. It accepts both paired and single end sequencing data. However, several FASTQ variants exist (eg. Illumina produces NGS data in FASTQ format with different quality score ranges -Illumina 1.3, llumina 1.5 and Illumina 1.8) and it would be tedious to support them all. To circumvent this inconsistency, the pipeline accepts only phred encoded FASTQ (Sanger FASTQ) files as input. Additionally, a reference sequence file in FASTA format is required which would be used as template to align the input datasets.

#### Pre-processing

The pipeline processes the input FASTQ files to assess and produce an overview of quality distribution of the dataset. Pre-processing enhances the quality of the input data by filtering and trimming reads. It filters off low quality reads followed by trimming low quality regions at the end of the residual reads. This pre-processing step is executed with a Perl script which reads the input data and considers the bases of reads as low quality, if the phred score [40] is less than 20. The script counts the number of low quality bases in a read and if the percentage of such bases is greater than 30%, the read is flagged as a low quality and discarded. Next, the average phred score at each position of the read and average phred score of whole dataset are computed independently. These scores are used as thresholds to trim the low quality bases of reads either from one end (5′ or 3′) or both the ends depending upon the average quality of bases at the ends. The threshold is set to 3 phred score units less than the average phred score. The number of bases to be trimmed from the ends is dynamically determined at runtime based on the sudden change of average phred scores greater than one phred score unit above the threshold. Paired end data is further processed by discarding all the reads not found in pairs. As a result, a FASTQ file with high quality reads is generated for downstream processing. Summary statistics of the pipeline describing the quality of raw data, trimming coordinates and the number of filtered reads is generated. The total run time to process the reads in this step depends on the amount of input data and the type of data (SE or PE). This pre-processing step is optional.

#### Sequence alignment

The pre-processed high quality reads are mapped onto the reference sequence. Alignment tools namely BWA [41], Maq [42], Bowtie2 [43], NovoAlign [44], and SOAP2 [45] have been embedded into the pipeline. The user could opt for any of the above mentioned tools and provides the option to change the default parameters. The default values for the parameters have been optimized and would be useful for users who are not familiar with the tools. This step starts with indexing of reference sequence for fast alignments followed by the mapping of the reads resulting in an alignment output in SAM format. By default Maq and SOAP2 alignments are in MAP format and were converted to SAM format using the Perl script “soap2sam.pl” [46]. The alignment statistics reported by the tools are available in the log file generated and the summary report of the pipeline. The alignment statistics provide details on the extent of mapping and guide the user on how the parameters affect the mapping process. Alternately, the SAM format alignment files generated from alternate mapping tools could be used for downstream processing in ISMU.

#### SNP identification

Good alignment with high read depth is an essential prerequisite with NGS data for efficient SNP discovery irrespective of sequencing platforms. The pipeline provides two different SNP calling methods namely: (i) SAMtools [47] and (ii) Coverage based Consensus Calling (CbCC) [48]. If the SAMtools method is selected, the SAM format files are converted to BAM format and bcftools used to call variations (between reference and samples) in VCF format [49]. The pipeline utilizes this information to find the confident polymorphism between the samples as well as between reference and samples. The SAMtools SNPs are further confirmed by the application of a custom filter as follows. The stack of bases at a given SNP position that satisfy a quality threshold of phred score >20 are derived. The frequency of the resultant bases is used to arrive at a consensus base. This consensus base could be a major base or an ambiguous base representing heterozygosity. These bases are then compared across genotypes to infer confident SNP. Resultant SNPs are reported with frequency information on the major base called and their read depth. In case SAM format alignments are not available by default, like when using Maq and SOAP aligners, SOAPsnp [50] and Maq (“cns2snp.pl”) are used instead of SAMtools respectively.

In the CbCC method, the pipeline reads the SAM files of samples and extracts the pileup information at all positions. This information was used to calculate and compare the consensus base or consensus allele for each sample at every corresponding position of reference. If the consensus base differs between the samples at that position then it is reported as an SNP. The pipeline computes the allele frequency at every position of the aligned sequence. If this allele frequency is above a *F_major_* threshold of 0.66, it is considered as a major/consensus base [48].

Finally, SNPs between the samples (pairwise) as well as against the reference sequence are reported. Additionally, a comprehensive non-redundant set of SNPs across all the samples as well as the reference is also reported as SNP genotyping matrix. All the reported SNPs are described with their position on reference sequence, major bases and the corresponding confidence ratio in each of the samples provided. These statistics further provide a confidence measure in selecting the SNPs for the design of genotyping assays.

#### Visualization

Although this step is optional, visualization of alignment and distribution of SNPs identified in samples would help highlight any possible sequencing errors or artefacts. The pipeline is integrated with two visualization tools namely, Tablet [51] and Flapjack [52]. The Tablet is used for visualizing alignment of reads on the reference sequence and to observe the called alleles at the SNP positions. It accepts SNP information in gff3 format to directly navigate to SNP positions on the alignment display [53]. The user has an option to select a set of SNP positions (*i.e.*, SNPs between any two samples) from the drop down list on the results window to view in Tablet. Flapjack is another Java based graphical genotyping software that could be used to visualize the SNPs or the allele distribution on each chromosome amongst the samples. A haplotype can be viewed and the user could also group, cluster or sort the genotypes/lines of similarity with other lines.

#### Input file for genotyping assay

This step of the pipeline generates an input file for the design of genotyping assays. The pipeline checks for various criteria for selecting SNPs suitable for genotyping. First, the SNP should have complete flanking sequences on either side. Second, the flanking sequences must not contain any ambiguous bases like “N”, “K”, “R”. Third, the flanking sequences should not contain any other SNP. An SNP satisfying these criteria are preferred for genotyping assay (However, flanking sequence length is different for KASP and Illumina genotyping platforms). All such potential SNPs are filtered to prepare a preliminary input file for genotyping assay development. One can exclude less informative SNPs either by visual inspection or on the basis of PIC value, so that highly informative SNPs could be used further in genotyping experiments.

### Pipeline output

The output generated by the pipeline include a summary report, SNP information for a pair wise combination of samples, amongst the samples, input files for KASP genotyping and preliminary input file for ADT scoring for Illumina genotyping. All the files are in a tab separated text file and a spread sheet with the file name “called_allele_data.xls” is also produced. Other than the log and vcf files, many other intermediate files like SAM and BAM files for each sample and filtered high quality FASTQ datasets are generated, which could be accessed in standalone ISMU. Input files could also be visualized.

### Evaluation of the pipeline

The pipeline was evaluated for its performance, accuracy and speed using two genomic and one transcriptomic datasets.

#### Pre-processing and alignment

The genomic dataset included whole genome re-sequencing (WGRS) data from four genotypes of chickpea (Pistol, Slasher, Hat Trick and Genesis 90) and the RAD sequencing data from ten genotypes of chickpea (ICCV 03107, ICC 4918, ICC 4930, ICC 4958, ICC 5270, ICCV 05530, ICC 5810, ICC 5912, ICC 6263 and ICC 8261). The transcriptomic dataset included RNAseq data from two peanut genotypes (HuaU12 and HuaU606). After pre-processing, the reads passing filter criteria in the WGRS, RAD and RNAseq datasets were 97.11% (125.07 million), 95.39% (74.22 million) and 98.18% (6.73 million) respectively. WGRS and RAD datasets were aligned to chickpea genome while RNAseq dataset was mapped against unigenes of peanut. On an average, 92.68% (Table 1), 90.5% (Table 2) and 80.61% (Table 3) of reads mapped onto the reference sequences from WGRS, RAD and RNAseq datasets respectively.

**Table 1:** Details about whole genome re-sequencing (WGRS) dataset used for evaluation of the pipeline.

Genotype name	Type	Total number of raw reads (PE)	Read length (bp)	Total number of filtered reads (PE)	Read length (bp) (post filter)	Alignment (%)	Number of SNPs as compared to the reference genome
Pistol	*desi*	33,467,106	101	32,193,245	86/87	93.03	317,991
Hat Trick	*desi*	32,021,614	101	31,126,432	86/87	92.53	156,255
Slasher	*desi*	31,093,427	101	30,286,838	86/88	92.51	351,844
Genesis 90	*kabuli*	32,210,496	101	31,467,878	86/88	92.66	253,472

Raw reads from above mentioned datasets were filtered and then aligned against the chickpea genome. These reads covered 92% of the reference genome. The pre-processing step of pipeline trimmed 100 bp reads into paired end reads of length 86 bp/87 bp each. The Hat Trick genotype showed half the number of SNPs called in comparison with other genotypes.

**Table 2:** Restriction site associated DNA (RAD) sequence dataset used for evaluation of the pipeline.

Genotype name	Total number of reads (SE)	Read length (bp)	Total number of filtered reads	Read length (bp)	Alignment (%)
ICCV 03107	2,360,400	100	2,250,687	78	91.27
ICC 4918	5,761,446	100	5,486,801	78	89.82
ICC 4930	10,595,164	100	10,103,218	78	89.90
ICC 4958	10,874,599	100	10,400,166	79	91.83
ICC 5270	8,198,607	100	7,790,524	78	90.38
ICCV 05530	8,011,084	100	7,611,453	78	89.96
ICC 5810	8,587,698	100	8,213,783	79	90.68
ICC 5912	5,422,669	100	5,183,888	79	91.12
ICC 6263	8,167,648	100	7,808,763	79	90.89
ICC 8261	6,245,558	100	5,943,309	81	88.94

Raw reads from above mentioned datasets were filtered and then aligned against the chickpea reference genome. The reads cover 88% to 92% of the reference genome. The pre-processing step of pipeline trimmed 100 bp reads into single end reads in the range 78 bp to 81 bp.

**Table 3:** RNAseq dataset used for evaluation of the pipeline.

Genotype name	Raw data		Filtered data		Alignment (%)	SNP with reference
	Total number of reads (PE)	Read length (bp)	Total number of reads (PE)	Read length (bp)		
HuaU12	6,857,839	90/90	6,733,549	72/74	82.51	41,225
HuaU606	6,771,173	90/90	6,649,229	72/74	78.71	44,984

Above mentioned RNA sequencing read data from two genotypes of peanut were included in this dataset. Raw reads were filtered and then aligned against the unigene sequences of peanut (ftp://ftp.ncbi.nih.gov/repository/UniGene/Arachis_hypogea/Ahy.seq.uniq.gz) as reference. The pre-processing step of pipeline trimmed 90 bp reads into paired end reads of length 72 bp/74 bp.

#### Variant detection

**Figure 2:**
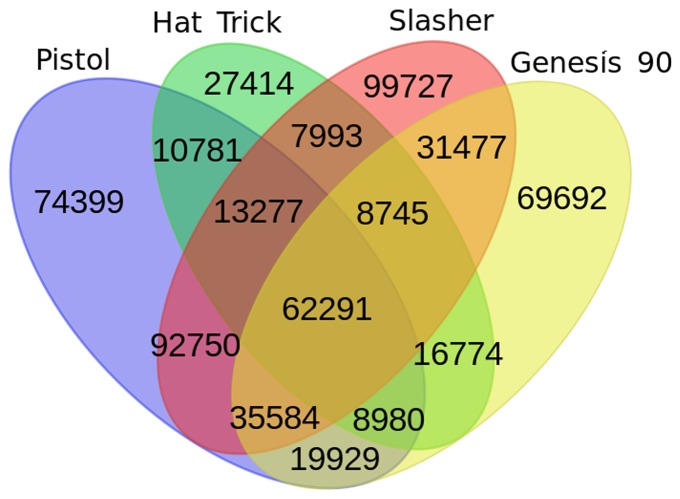
A snapshot on SNPs in four chickpea genotypes compared to the reference genome. The Venn diagram shows distribution of SNPs detected between four genotypes (Pistol, Hat Trick, Slasher and Genesis 90). The genotype CDC Frontier was used as a reference sequence. For instance, a total of 95,329 SNPs were found to be concordant between Pistol and Hat Trick genotypes. Similarly, amongst all the four genotypes 62,291 SNPs were found to be in common.

A total of 579,813 SNPs were identified from WGRS data (Figure 2) against the reference. A subset (62,291) of these SNPs in each genotype has shown polymorphism with reference genotype, CDC Frontier. Maximum variation (252,041 SNP) was found between Hat Trick and Slasher while minimum variation (145,415 SNP) was observed between Hat Trick and Genesis90. In case of RAD dataset, maximum number of SNPs with reference sequence was observed in ICC 4930. The total numbers of SNPs were observed to be in the range of 442 to 1151 between any pair of genotypes (Table 4). An SNP matrix consisting of 28,348 polymorphic positions across all the genotypes including reference was derived. Alleles in several genotypes were not found to be typed and hence they were regarded as missing due to no coverage or less coverage. This is a usual characteristic of RAD sequencing data and so one could opt for imputation to overcome this limitation [54]. In case of RNAseq dataset, HuaU606 shows more SNPs with reference than HuaU12 (Table 3). However a total of 13,294 SNPs were called between these two genotypes.

**Table 4:** Pairwise SNP distribution between genotypes identified in RAD dataset.

	ICCV 03107	ICC 4918	ICC 4930	ICC 4958	ICC 5270	ICCV 05530	ICC 5810	ICC 5912	ICC 6263	ICC 8261
**Reference**	5068	6250	9206	8502	7418	7461	8347	4985	6372	5664
**ICCV 03107**		442	470	599	501	499	528	455	667	471
**ICC 4918**			700	648	624	723	606	704	763	623
**ICC 4930**				1151	998	828	993	637	977	828
**ICC 4958**					829	1016	852	892	752	617
**ICC 5270**						945	886	791	783	613
**ICCV 05530**							972	778	924	774
**ICC 5810**								793	958	761
**ICC 5912**									910	743
**ICC 6263**										581

The genotypes show high variation with the reference when compared to pairwise combination of genotypes, indicating missing SNPs (a characteristic of RADseq) that could be imputed. Overall the numbers of SNPs between genotypes were found to be in the range of 442 to 1151.

### Profiling of ISMU

Allele calling is the most memory intensive part of the pipeline. A computational profile of the pipeline with the three datasets was recorded in order to observe the peak memory consumption, disk-space allocated and the time taken for the analysis to complete. In each case it was tested with 18 processors on a Linux based desktop machine with 48 Gb RAM (Table 5). The RNAseq dataset is found to be analyzed quicker than RAD followed by WGRS dataset. The reference sequences of RNAseq dataset are comparatively much smaller than the reference sequences used with WGRS and RAD datasets. It is apparent that more the input data, more time taken and hence more disk space required. The peak memory required was found to be on a higher side with WGRS and RAD datasets in contrast to RNAseq dataset. This is because the lengths of reference sequences in the case of RNAseq dataset are much smaller than WGRS and RAD datasets. In addition, WGRS and RAD datasets are 17 and 3 times larger than RNAseq dataset respectively and hence the memory requirement scales up accordingly. Interestingly RAD dataset consumes memory resources similar to WGRS inspite of being smaller in size to WGRS dataset. This is due to the fact that more number of genotypes are included in RAD dataset in comparison to WGRS dataset. Therefore we conclude that the number of genotypes as input, the reference chromosome size and the amount of data affect the RAM required the pipeline. Hence if one has more data and/or have more genotypes to be analyzed, we advise to run the ISMU pipeline accordingly on resourceful machines. However, transcriptome data or smaller genomes or a less number of genotypes of larger genomes could be efficiently handled on a desktop.

**Table 5:** Run time profile of the ISMU pipeline with three datasets (WGRS, RAD and RNAseq).

Datasets	WGRS	RAD	RNAseq
Method (aligner-SNPcaller)	BWA-samtools	Bowtie-samtools	SOAP2-CbCC
Total number of cores	18	18	18
Total number of genotypes	4	10	2
Input file size (Gigabytes)	105	19.4	6.2
Total time (hours)	26.25	4.5	2
Disk space (Gigabytes)	250	57	17.5
Peak memory (Gigabytes)	45	48	3.6

Three datasets (WGRS, RAD and RNAseq) were analysed independently with 18 processors on a 48 GB RAM Linux based machine. The disk-space used for analysis, peak memory used and the total time for the run were recorded. Analysis of RNAseq dataset was quicker than RAD/WGRS datasets owing to both small input size and smaller reference sequence pseudo-molecules/contigs. The disk space requirements were found to be proportionate to data size.

## Discussion

ISMU is a novel pipeline for SNP mining packed with unique features that are superior in comparison to other pipelines (Table 6). In general, the existing pipelines for SNP mining are command-line based which require technical expertise and therefore are not user friendly. Hence, ISMU includes a graphical user interface (GUI) to ease the analysis for the molecular breeder community as they are not familiar with technical nuances of Linux operating system and command line tools. Integration of robust pre-processing and visualization features especially flapjack for haplotype view makes it a unique pipeline of its own kind. Furthermore, the existing pipelines identify and report SNPs between reference and sample, however ISMU directly reports SNPs between two samples as well and provides a non-redundant list of SNPs amongst the samples. This could be used to compare and contrast allelic differences between the genotypes. The CbCC method for SNP detection is a reference free method useful for such plant species, where reference genome is not available. The detected SNPs were also provided as a spreadsheet containing heterozygosity, PIC value and allele distribution of each marker in different genotypes. This would help the breeding community to prioritize SNPs for genotyping. SNPs with high PIC values tend to show a high level of polymorphism in a given germplasm set which is desired for linkage mapping, QTL mapping and diversity studies, thus find applications in molecular breeding. In fact, the pipeline outputs the frequency of all the alleles present in samples and provides breeders to exploit this information for mining novel and rare allelic variants, which may have several functional significance for trait association mapping in crop plants. Minor allele frequency (MAF) for SNP locus can be inferred from the frequencies of the alleles directly. Alternately, lower value of heterozygosity at SNP locus provides a clue in finding locus with minor allele in the samples. Additionally, SNP data satisfying KASP genotyping platform criteria are filtered and provided in a KASP input format for conversion of SNPs to KASP assays. Similarly, SNPs satisfying criteria of the Illumina genotyping platform were also provided in a preliminary input format. This can be directly submitted to Illumina's online Assay Designing Tool (ADT) to score for predicting the success information and validation status [55]. Further if the markers qualify the threshold ADT score, they can be used for OPA (Oligo Pooled Assay) development on Bead Chips for different machine of Illumina (BeadXpress, Infinium Assay).

**Table 6:** Comparison of key features of the ISMU pipeline with similar pipelines.

Features	ISMU	SIMPLEX	ngs– backbone	GATK	inGAP	SeqGene	GAMES	TREAT	Atlas2
Free of charge	Y	Y	Y	Y	Y	Y	Y	Y	Y
SE/PE data handling	Y/Y	Y/Y	Y/N	Y/Y	Y/Y	n.m	Y/Y	Y/Y	Y/Y
NS/CS data handling	Y/N	Y/Y	Y/Y	Y/Y	Y/N	n.m	Y/Y	Y/N	Y/Y
Alignment	Y	Y	Y	N	Y	Y	N	Y	N
No. of alignment tools	5	1	1	N	2	n.m	N	2	N
Variant annotation	Y	Y	N	Y	N	Y	Y	Y	N
Highly customizable	Y	Y	Y	Y	N	Y	Y	Y	N
Homo−/heterozygosity	Y/Y	Y/Y	N/N	Y/Y	N/N	Y/Y	N/N	Y/Y	Y/Y
Quality reports	Y	Y	Y	Y	N	Y	N	Y	N
Graphical user interface	Y	N	N	N	Y	N	N	N	Y
Standalone	Y	Y	Y	Y	Y	Y	Y	Y	Y
HPC support	Y	Y	Y	Y	N	N	N	Y	N
Multi user support	Y	Y	N	N	N	N	N	N	N
Cloud support	N	Y	N	N	N	N	N	Y	Y

ISMU is one of the few tools that provide an easy to use graphical interface (GUI) packed with a wide choice of open source tools (alignment and variant calling) for handling NGS data. The information describing features of other pipelines is derived from Fisher et al. [60] and compared. The symbols “Y” and “N” represent, presence and absence of the feature in the pipeline. Numbers (1. 5, 2) indicate number of tool included in the pipeline. “n.m” refers to feature not mentioned.

In fact, this pipeline was initially developed for transcriptome data and later extended for genomic data. The pipeline has been designed to run on resource constrained environments (small laboratories) enabling NGS data to be processed even with very low amount of RAM. Installation of software and its dependency tools for processing NGS data and the downstream analysis are a daunting task for many researchers especially for a molecular breeder who may have little computational expertise. Therefore ISMU is also provided as a ready to use Virtual machine image facilitating the user to quickly get started on Desktop/Workstation and comfortably add more analytic power if required.

### Selection of tools

Open source NGS tools have been selected carefully and integrated in the ISMU. The pipeline presents the user with numerous options for alignment and SNP calling that makes it versatile and unique in comparison with similar pipelines. Multiple options help the user to reanalyze with different tools to find the best or concordant results. However each tool has its pros and cons. In case, one has high quality data available, the results with different tools would appear similar, but with low quality (shallow coverage) data the differences in results are evident [48].

Sequence alignment is one of the major steps that greatly affect the marker discovery. In this step the mapping tools use heuristics to align short reads to the reference sequence as exhaustive and accurate algorithms would not be computationally feasible. Initially, small regions (seed) in reads would be identified against the reference sequence where the location of the best match is most likely to be found. After identification of a smaller subset of possible mapping locations, slower but more accurate alignment algorithms such as Smith-Waterman are run on the limited subset [56]. Algorithms that are used to search the small set of potential alignments in the reference sequence can be broadly classified into two main categories - hash table and suffix tree/array. Hash based algorithms demand more resources (RAM) and computational time (ELAND/Maq/SOAP). Suffix arrays in combination with compression techniques like Burrow Wheeler Transformation (BWT) and FM index have been used to generate both space and time efficient alignment programs (BWA/BOWTIE/SOAP2), outperforming hash based methods [56], [57]. In total, one hash based software, Maq and four BWT based software, Bowtie2, BWA, NovoAlign and SOAP2 have been integrated into the pipeline. All tools are capable of handling short reads as well as SE and PE data; some software like BWA also capable for long reads. Hence it is advised to use BWT based software options in the pipeline because of quick run time and lower memory requirement.

The ISMU pipeline integrates four SNP calling programs. SAMtools (v0.1.19) [47] has been selected for variant calling as it is the most popular SNP calling program evident from the literature. SAMtools accepts alignment input in the SAM format produced by three alignment software namely BWA, Bowtie2 and NovoAlign. However, alignments using Maq and SOAP2 are not reported directly in SAM format, rather in Map format, but could be converted to SAM format. Map format files when converted to SAM format, does not contain header information. This header could be regenerated from the reference and supplied to SAMtools, to produce BAM files that could be used for SNP calling. However such BAM file does not work properly with SAMtools. Hence, as a workaround, Maqsnp (a script in Maq package) and SOAPsnp [50] software were integrated for consensus calling and to predict SNPs from and SOAP and Maq alignment respectively.

Alternately, another SNP calling method, Coverage based consensus calling (CbCC) [48] is included and could be opted to use with any of the alignment software integrated into ISMU. It uses alignment files in SAM format, either produced from alignment tools or converted into SAM format from other formats. On the other hand, CbCC has been written in Perl. The method generates a list of SNPs by directly comparing between two genotypes and has been very helpful in dealing with species lacking a finished reference genome sequence.

### Comparison with existing pipeline/software

Several open source pipelines for NGS data analysis are available for the research community. But not all of them support the analysis from raw NGS data. Very few pipelines like ISMU start with preprocessing of raw NGS data while pipelines such as GATK, GAMES, Atlas2 (Table 5) are designed to work starting with alignment files such as BAM/SAM. Many pipelines do not include the pre-processing steps which affects the false discovery rate of SNP predicted. However, in ISMU, the pre-processing step is an optional but recommended feature one could choose to avail depending on the quality of the input data. ISMU is a one-stop-shop tool for SNP analysis utilizing raw data as input with a user friendly interface (GUI) hiding the computational details. However, Atlas and inGAP are the only other pipelines with GUI. In fact ISMU is intended to assist genetics and breeding community who finds working with Linux/command line tedious. Therefore, a Virtual box image of the pipeline is provided to avert installation issues on windows as well Linux platforms. ISMU offers a wide range of alignment tools (Bowtie2, BWA, Maq, NovoAlign, and SOAP2) and SNP prediction methods (SAMtools, CbCC, SOAPsnp, Maq), a combination seldom available in other pipelines and hence is a unique feature in itself. In general the SNP calling tools provide SNPs with a quality/probability score, whereas ISMU provides a confidence ratio which reflects the read depth. This confidence ratio would be helpful in tagging high quality SNPs for genotyping studies. In fact, ISMU is the only pipeline which has an integrated facility for generating input file for developing assays for genotyping. The pipeline provides a non-redundant set of potential SNPs that could be used as an input file for KASP assay as well as Illumina based OPAs development for genotyping. While other pipelines provide SNPs against the reference only, ISMU in addition provides SNPs in pairwise combinations of genotypes as well as SNP matrix which is equivalent to the SNP matrix of GBS pipelines (TASSEL) [58]. ISMU can handle GBS/RAD data and produce the resultant matrix with PIC value and heterozygosity, another unique feature not found in other pipelines. Generally pipelines requires the user to execute a series of commands step-by-step for analysis but ISMU makes this process highly automated that the user needs to upload only the inputs and the results would be presented.

The pipeline is designed to handle genomic (WGRS or RAD) and transcriptomic datasets which include both SE (single end) and PE (paired-end) reads. To facilitate downstream analysis, SNP matrix or a matrix of called alleles for all genotypes is presented. The SNPs reported as a result were to be considered of high confidence. In WGRS dataset, the number of SNPs was observed to be very high with an average SNP density of 1.1/kb, while in RAD dataset SNP density was 0.05/kb owing to the nature of complexity reduction technologies which does not capture the complete variation. In RAD dataset, the extent of missing genotype information at the called position is huge and requires a better depth of sequencing or imputation of data. For alignment of RNAseq dataset, SOAP2 was used which neither allows gap nor clipping at the ends of reads at alignment. Because of this strict alignment mode, SOAP2 aligned lesser proportion of reads to distant reference sequences (unigene), evident in the low number of SNPs detected in comparison with gapped alignment based methods [43].

## Conclusion

ISMU is a validated SNP mining pipeline for NGS data from single or multiple samples. The prominent features of the pipeline include GUI, parallelization, multiple analysis tools, and multi-user support to facilitate analysis of huge NGS data for genotyping experiments. Although it has been developed for use with crop plants (e.g. chickpea, pigeonpea, etc.) it could also be used with animal and microbe data. Hence, the data could be processed independent of the organism. The easy to use GUI encourages non-bioinformatics scientists/researchers to analyse the data in their laboratories on the desktop machines. ISMU integrates proven NGS analysis tools and offers multiple options for alignment and SNP calling. It includes a complete workflow from pre-processing of raw data to deciphering SNPs and reporting markers towards genotyping experiments. The pipeline outputs variation information of the samples analyzed in a simple format for downstream analyses such as genotyping studies. Parallelization of ISMU enables efficient use of modern desktops with multiple cores. The complete application is rigorously tested and is also distributed on CDs as well as ready to use virtual box image.

ISMU targets plant molecular breeding community with little computational expertise. Plant breeders/researchers could potentially use it to quickly process the data and thereby obtain biological insights into genetic events. The SNPs detected by the pipeline could be used in applications such as linkage mapping, trait mapping, TILLING, GWAS and QTLSeq (Bulk segregant analysis).

## Materials and Methods

### Assembling tools

In order to develop an ISMU pipeline, open source tools such as BWA v0.6 (http://bio-bwa.sourceforge.net/bwa.shtml) [41], Maq v0.7 (http://maq.sourceforge.net/maq-man.shtml) [42], Bowtie2 v2.1.0 (http://bowtie-bio.sourceforge.net/bowtie2/index.shtml) [43], NovoAlign v2.07.13 (http://www.novocraft.com/main/index.php) [44], and SOAP2 v2.21 (http://soap.genomics.org.cn) [45] for alignment, SAMtools v0.1.19 (http://samtools.sourceforge.net) and CbCC v2.0 [48] for SNP calling, Tablet v1.12.03.26 (http://ics.hutton.ac.uk/tablet) [51] for visualization of SNP and Flapjack v1.12.05.25 (http://ics.hutton.ac.uk/flapjack) [52] for analysing allele distribution were downloaded. The details on development and their application of the above selected tools are provided in the above mentioned literature. Swings and AWT packages were used to develop GUI of the pipeline on Java (JDK 1.7) platform. Perl language v5.6 and bash scripting were used to add different functionality of pipeline. ISMU pipeline has been developed and tested on 64-bit Red Hat Linux based operating system (Cent OS 6) in Bash shell environment.

### Sequence data sets

In order to evaluate the performance of newly developed ISMU pipeline, two genomic datasets from chickpea and one transcriptomic plant dataset from peanut were collected. The first dataset comprises of the whole genome re-sequencing (WGRS) data from four genotypes of chickpea namely Pistol, Slasher, Hat Trick and Genesis 90, as reported in Varshney et al. [59]. The second dataset was the RAD sequencing dataset from ten genotypes of chickpea namely ICCV 03107, ICC 4918, ICC 4930, ICC 4958, ICC 5270, ICCV 05530, ICC 5810, ICC 5912, ICC 6263 and ICC 8261 [59]. These datasets are available at http://hpc.icrisat.cgiar.org/ISMU/datasets. The third dataset includes transcriptome data (RNAseq) from two peanut genotypes HuaU12 (SRR647081) and HuaU606 (SRR647076) downloaded from sequence read archive (SRA) database of NCBI. The WGRS and RNAseq datasets were paired end (PE) data while the RAD dataset was single end (SE). The WGRS and RAD datasets use the chickpea draft genome sequence [59] as a reference sequence while RNAseq dataset use peanut unigene representative sequences downloaded from the UniGene database (ftp://ftp.ncbi.nih.gov/repository/UniGene/Arachis_hypogea/Ahy.seq.uniq.gz).

### Development of the pipeline

Tools for alignment (BWA, Maq, Bowtie2, NovoAlign, and SOAP2), SNP calling (Samtools and CbCC), visualization of SNPs (Tablet, Flapjack) and analysing allele distribution (Flapjack) were decompressed and compiled on Redhat Linux. These tools were then integrated into a pipeline using Perl language. In this process, several in-house perl/bash scripts were written and embedded into the pipeline in order to bring automation, functionality and visualization. These scripts were used to provide required features such as pre-processing of data, create input files for Flapjack, processing SNP for calculating allele frequency, PIC value and generate files for developing Illumina and KASPar assays. Most of the scripts were written with multi-threading feature for speedy execution of the analysis for handling large datasets. In order to develop a GUI pipeline, interactive interface was developed related to data upload, selection of tools, customizing parameters for analysis, location of analysed data and output format. The input given by user through GUI is passed to backbone script of the pipeline which automatically generates a shell script to execute the different steps of pipeline. During the execution, shell scripted calls other softwares and embedded scripts in step wise manner while a GUI window shows the status of a run. In addition, it also provide an option to terminate the execution of pipeline at any stage. At the end of execution of pipeline, another page pops up showing options to download and display different outputs. The pipeline save all the output files in the user provided output folder which can be retrieved by the user at later stage.

### Assessment of the pipeline

Selected datasets were subjected to quality filtering in order to discard low quality reads i.e., reads having more than 30% of low quality bases (Q<20). These filtered reads were trimmed from both the ends which resulted in shorter read lengths. The resultant paired end reads would not be uniform in length. The reads passing these quality filters in the WGRS, RAD and RNAseq datasets were then aligned to a reference using different alignment tools in ISMU. WGRS dataset and RAD datasets were aligned to chickpea genome using BWA and Bowtie2 respectively. RNAseq dataset was mapped against unigenes of peanut using SOAP2. Samtools was used to call variants for WGRS and RAD datasets while CbCC was used with RNAseq dataset. All datasets were analysed on a 24 core Linux machine with 48 GB RAM.
